# Social Rhythm Therapies for Mood Disorders: an Update

**DOI:** 10.1007/s11920-016-0712-3

**Published:** 2016-06-24

**Authors:** Patricia L. Haynes, Devan Gengler, Monica Kelly

**Affiliations:** Department of Health Promotion Sciences, University of Arizona, 1295 N. Martin Ave., P.O. Box 245209, Tucson, AZ 85724-5209 USA; Department of Psychology, University of Arizona, 1503 E. University Blvd., P.O. Box 210068, Tucson, AZ 85721 USA

**Keywords:** Interpersonal and social rhythm therapy, Social rhythm, Depression, Bipolar disorder, Circadian rhythms, Sleep

## Abstract

Social rhythms are patterns of habitual daily behaviors that may impact the timing of the circadian system directly or indirectly through light exposure. According to the social rhythm hypothesis of depression, depressed individuals possess a vulnerability in the circadian timing system that inhibits natural recovery after disrupting life events. Social rhythm therapies (SRTs) support the implementation of regular, daily patterns of activity in order to facilitate recovery of circadian biological processes and also to improve mood. The majority of SRT research has examined interpersonal and social rhythm therapy (IPSRT) for bipolar disorder. Recent studies have examined IPSRT in inpatient settings, using alternative modes of delivery (group, combined individual and group, internet-based applications) and with brief timeframes. New forms of SRTs are developing that target mood in individuals who have experienced specific types of stressful life events. This manuscript reviews the theoretical and biological bases of SRTs and current literature on SRT outcomes.

## Introduction

Social rhythm therapy (SRT) was first conceptualized and developed by Ellen Frank [1] for outpatients with bipolar disorder. The therapy is based on the social rhythm hypothesis of depression [2], a theory that integrates research in the areas of life events and sleep/circadian rhythms. The social rhythm hypothesis is largely consistent with a conceptualization of bipolar disorder focusing on instability as proposed by Goodwin and Jamison [3]. The instability model posits that mood episodes arise from a combination of dysregulated neurotransmitter systems and a vulnerability within the circadian timing system. Social rhythm therapies promote stability of daily behaviors including sleep/wake in order to minimize the impact of disruptions to circadian rhythms.

In a nutshell, SRTs encourage individuals with dysregulated mood to develop and maintain moderately active and consistent daily routines. Social rhythm therapies target daily activity routines with the explicit goal of changing circadian biological processes that are contributing to the maintenance of dysfunctional biological and behavioral patterns. While these therapies support behavioral change in general, the key target is the client’s behavioral *pattern* that occurs across multiple days. In SRTs, clients develop a daily routine and monitor changes in their routine with a daily diary called the social rhythm metric [4].

Social rhythm therapy incorporates elements of behavioral activation and activity scheduling. In contrast to behavioral activation, SRTs do not specifically encourage increasing daily activities nor do they directly encourage engagement in activities that increase pleasure, mastery, or social engagement. Also, they do not directly encourage exposure to activities or stimuli that might be avoided. SRTs encourage engagement in a moderate amount of activities at a habitual time each day in order to promote stability. Social rhythm therapies do not explore values nor the meaning of interpersonal life transitions or severe life events beyond the impact these events may have on a person’s daily routine. Social rhythm therapies are informed by our knowledge of the circadian clock. Thus, the key mechanism in SRTs is biological. For this reason, SRTs have typically been combined with other theoretical psychotherapies to provide a psychological framework by which to support changes in daily routine and recovery after disrupting life events.

The purpose of this manuscript is to briefly describe the theoretical basis for social rhythm therapies and the social rhythm hypothesis of mood disruption, informed by principles of the circadian timing system. In addition, we will describe how SRTs have been implemented and combined with other psychological approaches. Finally, we conclude with an evaluation and synthesis of the most recent literature in SRTs.

## Social Rhythms and Circadian Timing System

A variety of biological and behavioral processes occur in regular, daily rhythmic cycles, or circadian rhythms. Circadian (from *circa* meaning “approximately” and *dies* meaning “day”) rhythms are patterns of various physiological processes that vary systematically across the 24-h period. For this reason, the circadian organization of physiological events has also been termed predictive homeostasis; corrective physiological responses are initiated in anticipation of a predictably timed event (e.g., physiological changes leading to wakefulness with sunrise) [5].

Circadian rhythms are direct expressions of the circadian clock, or central circadian oscillator [6], that is positioned in the superchiasmatic nucleus (SCN) of the hypothalamus. The oscillator generates circadian rhythms in a variety of biological variables. The most common circadian rhythm variables studied in human physiology are core body temperature, cortisol, and melatonin. Sleep and wakeful activity are also considered expressions of the circadian oscillator.

In order to live in our environment, the circadian clock must become entrained to a 24-h cycle. Entrainment refers to the process by which the internal circadian clock becomes synchronized with the external environment. Light is the most powerful external time cue, or *zeitgeber* (German for “time giver”), in that the day-night cycle entrains internally generated rhythms of sleep-wake, body temperature, and neuroendocrinological processes [7]. In general, zeitgebers are regular, external occurrences that synchronize the internal clock to environmental cues. Some research supports the presence of non-photic zeitgebers [8–10], especially when light is low. Social zeitgebers are personal relationships, social demands or tasks, or routinized activities that are theorized to impact the timing of biological rhythms [2]. Examples of social zeitgebers include awakening in the morning, eating meals, exercising, and other activities within typical daily routines. In this respect, social is broadly defined as any type of interaction with the environment; thus, even solitary activities performed routinely are considered social zeitgebers for the circadian clock.

Circadian rhythms research has advanced significantly in the last two decades with the advent of forced desynchrony protocols [11, 12] and the related ability to disentangle sleep and other activities from endogenous circadian rhythms. From these developments, it has become increasingly clear that social zeitgebers may not be zeitgebers at all. Instead, social zeitgebers can best be conceptualized as behaviors that modify the expression of biological rhythms by altering exposure to light. Human behavior does not typically occur in the context of constant darkness, and the original studies examining the impact of social stimuli on the circadian timing system did not control for dim levels of light (see Mistlberger and Skene for review [13]). The circadian clock is sensitive to dim light, especially at certain times of day and with prolonged exposure to dim light. In studies where light exposure was controlled, sleep-wake schedules and social cues had a negligible impact on the circadian timing system (see Mistlberger and Skene for review [13]). However, alterations in sleep-wake schedules and social cues modify exposure to light and this change in light exposure can disrupt the timing of circadian rhythms. We see examples of this effect in night shift workers, travelers with jet leg, and other individuals who have sleep/wake schedules that are not consistent with the natural light-dark schedule.

Social rhythms refer to the *pattern* in which daily, habitual behaviors (“social zeitgebers”) occur (e.g., eating breakfast at 8 a.m. on one day and 11 a.m. the next, versus 8 a.m. each day), rather than the frequency of the behaviors (e.g., eating breakfast every day) or the type of behaviors (e.g., pleasurable vs. routinized). Social rhythms are typically measured by the social rhythm metric (SRM) [4, 14], a daily diary that is administered over 2 weeks. The SRM index assesses the variability of 17 daily, habitual behaviors. Studies have indicated that the following five behaviors account for the majority of variability in daily routine: (1) get out of bed; (2) first contact (in person or by phone) with another person; (3) start work, school, housework, volunteer activities, and child or family care; (4) have dinner; and (5) go to bed. Normative data collected from healthy, never-depressed participants indicates that the SRM index (stability in daily routine) increases with age, suggesting that lifestyle regularity may be an adaptive response to age-related biological changes in the circadian system [15].

## Social Rhythm Hypothesis of Depression

According to the social rhythm hypothesis of depression, stressful life events interrupt a person’s daily routine or regular exposure to “social zeitgebers.” This disruption then leads to instability in specific biological rhythms, such as sleep, in vulnerable individuals [2]. This instability is also thought to lead to mild somatic symptoms in non-vulnerable individuals. Vulnerable individuals may be unable to reverse the disruption and instead maintain a pattern of instability. Ehlers and colleagues [2] suggested that the vulnerability factors may be biological/genetic in origin, such as a family history of an affective disorder, a low threshold to rhythm disruption, a longer recovery period for rhythm stabilization, or a stable system process that sustains abnormal rhythms after initial disruption. The social rhythm hypothesis was modified by Haynes and colleagues [16] to incorporate light exposure as a significant factor.

Numerous studies provide support for the social rhythm hypothesis. Outpatients with current depression have less consistent social rhythms than normal controls [16–18], and one study suggests this relationship is mediated by less ambient light exposure [16]. In addition, social rhythm inconsistency was associated with a shorter time to an affective episode in undergraduates with bipolar spectrum disorders [19] and the development of bipolar spectrum disorder in high reward-sensitivity adolescents [20]. Researchers also found that life events associated with disruptions in social rhythms (e.g., overseas travel, being fired from a full-time job, marital separation) were better predictors of manic episodes than severe life events in general in a sample of patients with bipolar disorder [21]. Social rhythm disruption (SRD) events were also associated with worse sleep in depressed outpatients [22] and more sleep loss in undergraduates with bipolar spectrum disorder [23] as compared to normal controls. Findings are mixed as to whether SRD events predict the onset of depressive episodes in patients with bipolar disorder [21, 24]. One reason for the inconsistency in findings may be due to the study of bipolar patients on pharmacotherapy, as this leads to the natural self-regulation of daily routine [25].

Only a few studies have investigated social rhythms in other mental disorders and conditions. Similar to individuals with major depressive disorder, individuals with anxiety disorders have fewer habitual behaviors and less consistent social rhythms [26]. Shear and colleagues suggested that individuals with anxiety disorders might have a heightened sensitivity to events that disrupt social rhythms and that irregularity in daily routine may contribute to the sense of unpredictability and uncontrollability that is characteristic of these disorders [26]. In addition to anxiety, individuals with insomnia disorder were more likely to have lower (more irregular) social rhythms than normal sleepers [27]. One epidemiological study conducted in Russia, the USA, and Germany (*n* = 8095) found that self-reported lifestyle irregularity was associated with greater health problems, depression, anxiety, and stress [28]. This study was limited by the use of a new measure for social rhythms that did not use time-based monitoring and instead queried individuals about the irregularity of 10 different activities.

## Summary

Social rhythms refer to the day-to-day variability of daily, habitual behaviors. They are most commonly measured by the SRM. Social rhythms were originally conceived based on the concept of social *zeitgebers*. More recent research suggests that light exposure is the primary zeitgeber for the circadian clock. Nonetheless, social rhythms may affect circadian timing indirectly by modifying exposure to light; they may also directly affect the expression of various circadian rhythms. Cross-sectional and prospective studies support that social rhythm instability is an important risk factor for mood disruption. The majority of research examining social rhythms in psychiatry has occurred within the context of bipolar spectrum disorders. Research is only beginning to assess social rhythm stability in other populations, including individuals with anxiety and insomnia disorders.

## Social Rhythm Therapies, Randomized Controlled Trials (RCTs)

Four randomized controlled trials have examined social rhythm therapies for bipolar disorder, all within the context of interpersonal and social rhythm therapy (IPSRT) (see Table 1). Interpersonal and social rhythm therapy combines SRT with interpersonal therapy. Interpersonal therapy is an efficacious therapy for unipolar depression. Individuals in interpersonal therapy learn to resolve disrupting life events relating to the problem areas of grief, role disputes, role transitions, or interpersonal deficits [29]. In total, three randomized controlled trials have examined IPSRT as an adjunct to medication, and one RCT has examined IPSRT as a primary treatment.

**Table 1:**
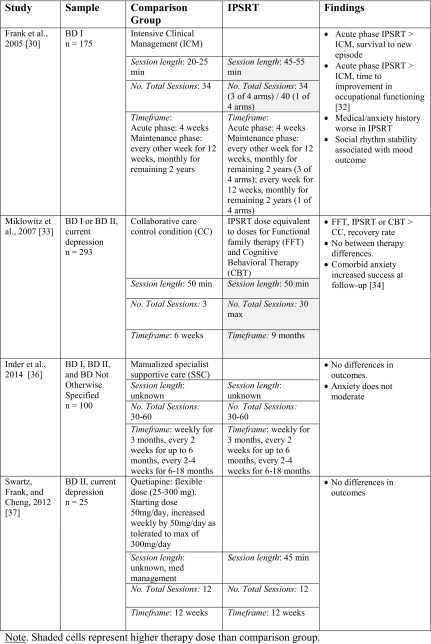
Randomized controlled trials (RCTs) on interpersonal and social rhythm therapy (IPSRT)

Frank and colleagues [30] compared IPSRT to intensive clinical management (ICM) in bipolar disorder I (BD I) patients in a cross-over design over the course of 4 weeks (acute phase) and 2 years of maintenance treatment. They found no difference between groups in the time it took to stabilize symptoms. Compared to participants who received ICM in the acute phase, participants who received IPSRT in the acute phase lasted longer without a new mood episode (technically a mood relapse, see Hollon and Ponniah [31] for discussion). Interestingly, this finding was not consistent for individuals with significant comorbid medical or anxiety disorders. Participants assigned to IPSRT in the acute phase also exhibited more rapid improvement in occupational functioning than those assigned to ICM [32]. In addition, IPSRT was associated with higher levels of social rhythm regularity at remission, and higher levels of social rhythm regularity at remission were associated with a lower likelihood of recurrence [30]. Thus, social rhythm stability was likely a mechanism of the therapy accounting for mood relapse.

Interpersonal and social rhythm therapy was also examined with functional family therapy (FFT), cognitive behavioral therapy (CBT), and a collaborative care control condition (CC) in the multi-site Systematic Treatment Enhancement Program for Bipolar Disorder (STEP-BD) effectiveness psychotherapy trial (*n* = 293 participants) [33]. Final outcomes suggested that individuals in the more intensive psychotherapies (FFT, IPSRT, and CBT) had a faster (113 days) and more successful recovery rate (62 %) compared to individuals in CC (146 days, 52 %). There were no statistical differences among the three intensive conditions, although 77 % of participants in FFT recovered compared to 65 % of participants in IPSRT and 60 % in CBT. Recent follow-up analyses from this study [34••] indicated that depressed participants with BD I or II and one lifetime anxiety disorder were more likely to recover with intensive psychotherapy (IPSRT, CBT, and FFT) than participants without anxiety or participants with more than one anxiety disorder. Follow-up analyses suggested bipolar participants with lifetime posttraumatic stress disorder or generalized anxiety disorder were the most likely to benefit from intensive intervention compared to CC. As stated above, Frank and colleagues [30] found that an anxiety disorder history was associated with *worse* response to IPSRT; however, subset analyses from their sample suggested that panic spectrum symptoms were associated with longer times to remission [35]. These findings indicate that the type of anxiety is a potentially important moderator in IPSRT response.

A significant limitation of both of these studies is that individuals in IPSRT received much more attention from a clinical provider than individuals in the control conditions. Bipolar participants in the therapy conditions in STEP-BD could receive up to 9 months/30 sessions of therapy compared to individuals in collaborative care who only received 1.5 months/3 sessions of counseling. In contrast, Inder and colleagues [36••] conducted a randomized, controlled trial comparing IPSRT to manualized specialist supportive care (SSC) in medication-stable adults and young adults with bipolar disorders (BD I, BD II, and BD Not Otherwise Specified). The SSC was a control condition with equivalent therapy time and clinical attention as IPSRT. Both groups improved on outcome measures, but there were no differences between IPSRT and SSC on cumulative depression and social functioning at 1 year post-baseline assessment. There were also no moderating effects of age, substance use disorder, or anxiety disorder comorbidity.

Finally, a randomized pilot study (*n* = 25) conducted by Swartz, Frank, and Cheng [37] compared the effects of IPSRT to quetiapine, an atypical antipsychotic, for the acute treatment of BD II depression. Currently depressed, non-medicated participants with BD II were randomly assigned to 12 weekly sessions of IPSRT or a flexible dose (25–300 mg) of quetiapine. Although both groups showed declines in depression and manic symptoms, there were no significant differences between groups on therapy response. Treatment satisfaction was high in both groups, and treatment preference had no effect on outcomes.

## New Applications, IPSRT

Several recent studies have evaluated IPSRT for mood disorders in a group format or a format combining group and individual therapy. Swartz and colleagues [38] investigated the practicality and effectiveness of implementing group IPSRT in inpatient, outpatient, and intensive outpatient programs at an academic medical center. IPSRT was tailored to meet the needs of inpatient therapy by focusing on a range of diagnoses, emphasizing the stabilization of social rhythms even during brief stays, and providing a 2-day training to all inpatient staff. A manual detailing each intervention was created to ensure consistency. The authors concluded that IPSRT was feasible across all three levels of care as shown by declines in depressive symptoms in the outpatient and intensive outpatient groups and an increase in group attendance in the inpatient group. Future controlled studies are necessary to extend this line of research. In particular, research should examine whether IPSRT is efficacious in reducing symptoms, length of stay, and readmission in hospitalized psychiatric patients.

Next, Hoberg and colleagues [39] adapted IPSRT in order to provide a treatment option for individuals unable to participate in weekly sessions. Seven outpatients with BD I or BD II completed two 60-min individual IPSRT sessions followed by six 60-min group IPSRT sessions with an advanced practice psychiatric nurse over the course of 2 weeks. In the individual sessions, the therapist reviewed the patient’s course of illness and interpersonal problem areas and developed target activity times. Group sessions focused on stabilizing daily routines and improving interpersonal relationships. Depressive symptoms and psychosocial functioning improved significantly at the 12-week follow-up assessment, although only 16 % of the sample met remission criteria for depression symptoms. There were no significant differences from baseline to 12 weeks on mania or BD severity. Participants reported high satisfaction with IPSRT administered in this format [39]. Results from this study must be qualified by the small sample size and lack of control group.

A third pilot study evaluated IPSRT in a group format for 22 outpatients with BD [40]. In this study, IPSRT consisted of 16 total, 2-h weekly sessions over the course of 1 year. Sessions were divided with the first half focusing on IPT and the second half focusing on SRT. Findings revealed no significant change in manic symptoms. However, patients did experience a significant reduction in depressive symptoms and a stabilization in social rhythms from pre- to post-treatment. The researchers did not examine whether change in social rhythms was associated with depression response. Taken together, these three studies suggest that group IPSRT may be as effective as individual IPSRT for depression symptoms. Future RCTs are necessary to examine whether group IPSRT is efficacious by employing an active attention control group design.

Previous results from a small, open trial suggest that IPSRT modified for adolescents with BD is associated with a reduction in manic and depressive symptoms and an increase in global functioning [41]. Goldstein and colleagues [42•] built upon this work by conducting a pilot study of IPSRT in adolescents with a family history of BD (*n* = 13 at risk adolescents). They administered a total of 12 sessions of individual IPSRT over 6 months. Therapy included varying degrees of parental involvement, depending on treatment phase and developmental level. Adolescents and their families reported high satisfaction with IPSRT and less oversleeping on the weekends. Pre-treatment assessment indicated only mild symptoms on the depression and mania rating scales, and there was no change in symptoms over time. Clinician ratings of psychosocial functioning showed minimal improvement over time. Interestingly, 67 % of families contacted by staff declined therapy based upon the adolescent’s belief that they did not need therapy, indicating that IPSRT may not be an acceptable prevention option for adolescents.

## New Modifications, SRTs

Within the last 3 years, several new versions of SRTs have emerged. Pfoff, Zarotney, and Monk [43•] conducted a pilot RCT (*n* = 38) comparing function-based therapy (FT) to an active attention control therapy (CT) that concentrated solely on emotional problems in bereaved older adults. Function-based therapy focused specifically on improving daily lifestyle regularity. Similar to IPSRT, the therapist and participant worked towards stabilizing daily schedules and implementing healthy sleep practices using the SRM. Individuals in both conditions received 10 sessions of therapy over 6 months (weekly for the first month, biweekly for the second month, and monthly for the next 4 months). Functional and emotional realms were assessed using measures of grief, depressive symptoms, sleep quantity, and sleep quality. Both therapy groups improved on all measures from baseline to post-treatment, but individuals in the FT group showed significantly greater improvements on depression symptoms, time spent asleep, and sleep efficiency (percentage of time in bed spent asleep) than those in the control condition. All sleep items were assessed by the Pittsburgh Sleep Quality Index (PSQI) [44]. A larger-scale RCT utilizing prospective, gold-standard sleep diaries is warranted to examine the efficacy of FT and whether treatment gains are sustained over time.

Lieberman, Swayze, and Goodwin [45] conducted a pilot study evaluating an internet-based application, MoodChart, that assists patients with bipolar disorder in stabilizing their social rhythms. Using this program, a total of 64 participants who reported having been diagnosed with bipolar disorder recorded the times they performed daily activities and their mood at the time of data entry. They were then asked to set target times for their activities and attempt to begin each activity within 45 min of the chosen target time. Participants were given feedback about their success by displaying a “hit rate,” a percentage of time each activity was performed within 45 min of the target time within the past 7 days. At posttreatment, participants had a 31 % increase in social rhythm stability and a small, but significant decrease in mood severity as assessed by the National Institute of Mental Health Life Chart Methodology. Future randomized controlled trials are necessary to determine whether MoodChart is efficacious for BD.

Finally, Haynes and colleagues [46•] expanded on previous studies examining SRT in groups by testing a 12-week group cognitive behavioral social rhythm therapy (CBSRT) for 24 veterans with posttraumatic stress disorder (PTSD), depression, and sleep problems. Cognitive behavioral social rhythm therapy integrates SRT and cognitive behavioral therapy for mood and sleep disturbances. Cognitive therapy in CBSRT was employed to help individuals correct misattributions about their somatic or internal state (e.g., fatigue) and did not directly address trauma-related symptoms [47]. It was also employed to dispute dysfunctional cognitions that interfered with the maintenance of consistent social rhythms. Veterans improved on all measures with large effect sizes from baseline to 3-month follow-up on PTSD symptoms, depressive symptoms, sleep quality, number of awakenings, sleep onset latency, and nightmare frequency. Interestingly, improvement in social rhythm stability was associated with improvement in PTSD but not depressive symptoms. Results from a randomized controlled trial are pending.

## Conclusions

Altogether, recent studies suggest that IPSRT is feasible and satisfactory to patients with bipolar disorder. While some studies indicate that IPSRT is helpful in improving mood symptoms and preventing an episode of BD, the strength of these effects is unclear. IPSRT is currently considered a possibly efficacious treatment [31]. Randomized controlled trials employing an active control condition are necessary to determine whether IPSRT is more or less beneficial than other therapies for BD. Both Miklowitz et al. [33] and Inder et al. [36••] found very few differences in outcomes between active attention therapy comparison conditions when time and attention were controlled. To be classified as efficacious, treatments must have at least two between-group experiments demonstrating efficacy through treatment superior to pill, placebo, or other treatment and/or are equivalent to an established treatment [48].

In addition, future trials should include estimates of clinical significance and ensure that follow-up assessments are conducted to assess whether treatment gains are sustained. The SRM plays an intrinsic role in SRT administration. Thus, investigators examining SRTs should report data examining whether changes in social rhythms correlate with clinical improvement in mood, sleep, and other circadian measures, as this would provide valuable information about treatment mechanism.

Studies have begun to examine whether social rhythm therapies are effective in other populations that have faced severe life events, including bereaved seniors and veterans with PTSD. Future research could benefit from extending this line of research and examining whether comorbid anxiety or stress-related disorders are potential moderators of treatment response. A well-established research base has shown that life events predict onset and reoccurrence of major depressive disorder [49]. Given this, it is surprising that no studies have investigated whether SRTs are effective for treating or preventing unipolar depression. Much more work may be done in this area, especially within a prevention framework, in order to determine whether SRTs promote resiliency after disrupting life events.
